# Transport Stress Induced Cardiac NO-NOS Disorder Is Mitigated by Activating Nrf2/HO-1/NQO1 Antioxidant Defense Response in Newly Hatched Chicks

**DOI:** 10.3389/fvets.2022.938826

**Published:** 2022-06-10

**Authors:** Hao-Liang Xu, Hui Li, Rong-Kun Bao, Yi-Xi Tang, Ahmed Ibrahim Ahmed Elsherbeni, Hassan Bayoumi Ali Gharib, Jin-Long Li

**Affiliations:** ^1^College of Veterinary Medicine, Northeast Agricultural University, Harbin, China; ^2^Laboratory of Sport Physiology and Biochemistry, Harbin Sport University, Harbin, China; ^3^Animal Production Research Institute, Agricultural Research Centre, Giza, Egypt; ^4^Animal Production Department, Faculty of Agriculture, Cairo University, Cairo, Egypt; ^5^Heilongjiang Key Laboratory for Laboratory Animals and Comparative Medicine, Northeast Agricultural University, Harbin, China; ^6^Key Laboratory of the Provincial Education, Department of Heilongjiang for Common Animal Disease Prevention and Treatment, Northeast Agricultural University, Harbin, China

**Keywords:** transport stress, newborn chicks, oxidative stress, NO metabolism, Nrf2 signaling pathway

## Abstract

With the development of the intensive poultry industry, the health problems of chickens caused by transportation have attracted more and more attention. Transport stress reduces performance, immune function, and meat quality in chicks, which has become one of the most important factors that endanger the development of the poultry industry. Currently, studies on the effects of transport stress have mainly focused on the performance of livestock and poultry to be slaughtered. However, the effects of transport stress on heart damage and oxidative stress in newborn chicks have not been reported. In this study, we selected newborn chicks as the object. This study was intended to explore the effects of transport stress on the heart damage of newly hatched chicks. The findings suggested that transport stress could cause oxidative stress in the hearts of newly hatched chicks by increasing the levels of malondialdehyde (MDA), hydrogen peroxide (H_2_O_2_) and decreasing the contents of Total antioxidant capacity (T-AOC), and the activities of antioxidant enzymes (SOD), together with increasing the activities of antioxidant enzymes (Catalase (CAT) and Glutathione S-transferase (GST)). Transport stress disrupted the balance between oxidation and antioxidant systems. The Nrf2 signaling pathway was activated by transport stress and triggered the transcription of antioxidant signaling. In short, transport stress-induced nitric oxide (NO)—nitric oxide synthases (NOS) system metabolic disorders and cardiac oxidative stress are mitigated by activating the nuclear factor-erythroid 2-related factor 2 (Nrf2)/heme oxygenase-1 (HO-1)/NAD(P)H quinone oxidoreductase-1 (NQO1) antioxidant defense response in newly hatched chicks.

## Introduction

The economic losses caused by transport stress are enormous for livestock and poultry. Transport stress is an animal syndrome caused by a series of stress factors (1). There will inevitably be disturbances such as acceleration, bumps and noise during transportation, and it will change the original schedule and diet during the transport. The transport environment is different from the barns, squeezing and circling stimulate newborn chickens in the warehouse (2). In the process of transportation, broiler and layer chicks suffer from multiple severe stimuli, such as loading and unloading of chicks during transportation (3). Although the faint and transient stimulation will help animals adapt to changes and enhance physical fitness, but the transport stress syndrome occurs beyond a certain limit, which may manifest as cardiac dysfunction, organ failure, shock, or even death (4, 5). Recent reports document the changes in adult poultry under transportation, nevertheless, the probable effects of transportation on heart damage of newly hatched chicks are not yet fully clear.

Transport stress leads to weight loss and aggressive behavior in chicks, causing the production rate of laying hens to drop or stop (6). Transport stress can induce excessive accumulation of reactive oxygen species, leading to oxidative stress and cell damage (5). Nuclear factor-erythroid 2-related factor 2 (Nrf2), an oxidative stress responder, plays a considerable role in defense mechanisms (7). The Nrf2 signaling pathway has a high degree of oxidative-inducing sensitivity and is capable of pleiotropic regulation of basal expression and inducible expression of phase II metabolic enzyme genes, thereby exerting antioxidant and cytoprotective effects (8, 9). If Nrf2 is deleted or activated, it will aggravate the toxicity of oxidative stress to cells, causing cell dysfunction or initiating apoptosis program (10). Nrf2- heme oxygenase-1 (HO-1)—NAD(P)H quinone oxidoreductase-1 (NQO1) signaling pathway is a cardinal channel to activate the expressions of phase II enzymes and antioxidants (11), and numerous natural compounds demonstrate their activities through it. The Nrf2- HO-1- NQO1 signaling pathway inhibits the cell defenses and results in oxidative stress, which could be considered as an out-off-balance of oxidants and antioxidants (12, 13).

The evidence provides convincing data that the implication of nitric oxide (NO) in oxidative stress processes within the NO-NOS system (14). NO is generated by three dissimilar isomers of nitric oxide synthases (NOS) (15), which alter L -arginine to L -citrulline with the consequent free of NO: the neuronal NOS (nNOS) and the endothelial NOS form (eNOS) are modulated by Ca^2+^ activated calmodulin, whereas the inducible isoform NOS (iNOS) is calcium-independent (16). Considering the crucial part of the NO/NOS system in these cases, we speculated that the effects of transport stress on the heart of newly hatched chicks were related to oxidative stress. As is known to all, the healthy chicken is the beginning point for well production performance of poultry, nevertheless, the effects of transport stress on chicks are little known.

Therefore, the purpose of this study is to clarify the effect of transport stress on the heart damage of newborn chicks and the metabolic disturbance of the NO-NOS system, and to determine the adaptive response to transport stress by activating the antioxidant defense induced by Nrf2. However, it is not clear how transport stress causes heart damage in chicks.

## Materials and Methods

All procedures adopted in the current study were implemented in accordance with the ethical protocols set by the Institutional Animal Care and Use Committee of Northeast Agricultural University and the animal welfare regulations.

### Experimental Design and Tissue Collection

A total of 180 newborn chicks (Hy-Line Variety White) were purchased from the Xiang Fang Chicken Farm (Harbin, China) and were split into the control group, transport group, and simulation transport group. Chick transport crates were used during the transport stress. A separate crate was employed for each repeat group. The model of transport stress and simulated transport stress was designed as described in the former study (4). Brief, the chicks in the simulation transport group were placed in an electric cradle (50 cycles/min, swinging back and forth; 28°C and 50% relative humidity) for 2, 4, and 8 h, but the chicks in the control group were placed in a quiet electric cradle (0 cycle/min; 28°C and 50% relative humidity). The chickens in the transport group were transported on the road by a truck for 2, 4 or 8 h, with a distance of about 480 km and an average speed of 60 km/h. The chicks were not allowed *ad libitum* to feed and water. After the stress-treated, the chicks were euthanized with carbon dioxide. Each animal's heart was quickly removed and rinsed with ice-cold sterile phosphate buffer saline; it was then immediately frozen in liquid nitrogen and stored at −80°C for further analysis.

### Determination of the Serum Creatine Kinase Content of Newborn Chicks (CK Activity)

The whole blood was centrifuged at 2,500 g for 10 min to obtain serum. The Creatine kinase (CK) determination is based on the continuous detection method recommended by the German Association for Clinical Chemistry and the International Federation of Clinical Chemistry. It is passed on a modular P analyzer (Hitachi, Tokyo, Japan) through commercial reagents (Roche Diagnostics, Mannheim, Germany) CK-N-acetylcysteine kinetics measurement (37°C) was measured.

### Histopathological Assessment

The hearts of the control, transport, and simulation transport group were fixed in 10% neutral buffered formalin and processed using routine histological techniques. After paraffin embedding, they were cut into 5 μm sections and stained with hematoxylin and eosin to evaluate the tissue structure. Visualize the slides using an optical microscope (Nikon, Japan). Throughout the process, the image acquisition parameters and microscope settings remain the same.

### Determination of Antioxidant Function of Newborn Chicks Heart

Malondialdehyde (MDA) content was measured according to the manufacturer's instructions using a commercial kit (Nanjing Jiancheng Bioengineering Institute, A003-1, Nanjing, China). In short, MDA was determined in the absence of light by using 35% (p/v) of 1 ml of trichloroacetic acid. After that, add 1 ml of 0.5% thiobarbituric acid and heat the sample to 80°C for 30 min. The sample was then cooled in an ice bath, centrifuged and the absorbance of the supernatant was determined at 532 nm. The calibration curve of MDA solution was used to estimate lipid oxidation, while MDA level was expressed as per mg of protein (nmol MDA/mg prot).

The content of hydrogen peroxide (H_2_O_2_) was measured with an H_2_O_2_ commercial kit (Nanjing Jiancheng Bioengineering Institute, A064, Nanjing, China) according to the manufacturer's instructions. A complex cover H_2_O_2_ and the chromogenic agent, and the content of H_2_O_2_ can be calculated by measuring the absorbance at 405 nm.

Total antioxidant capacity (T-AOC) was measured with a T-AOC detection kit according to the manufacturer's instructions (Nanjing Jiancheng Bioengineering Institute, A0015-1, Nanjing, China) by converting Fe^3+^ into Fe^2+^; the phenanthroline substance forms a complex, which can be measured with a spectrophotometer.

Total superoxide dismutase (T-SOD) activity in the homogenate was assayed by a xanthine oxidase method at 550 nm (Nanjing Jiancheng Bioengineering Institute, A001-1, Nanjing, China). One unit of superoxide dismutase is defined as the amount of enzyme that causes 50% inhibition of pyrogallol.

Glutathione S-transferase (GST) was measured with a GST detection kit according to the manufacturer's instructions (Nanjing Jiancheng Bioengineering Institute, A004, Nanjing, China). GST catalyzes the binding of glutathione to 1-chloro-2,4-dinitrobenzene. GST activity can be calculated by measuring the rate of increase in absorbance at 340 nm wavelength.

Catalase (CAT) activity was spectrophotometrically measured according to the manufacturer's instructions (Nanjing Jiancheng Bioengineering Institute, A005-1, Nanjing, China), based on the reaction of catalase and methanol, in the presence of the optimal concentration of H_2_O_2_, the generated formaldehyde can be colorimetrically quantified at OD 540 nm.

### Quantitative Real-Time Polymerase Chain Reaction

Total RNA was isolated from every heart tissue sample using TRIzol reagent according to the manufacturer's specifications (Beijing Tiandi, Inc., Beijing, China). The RNA extraction, quantitative real-time polymerase chain reaction (qPCR) procedure, and quantification method used were identical to those previously described. Quantitative real-time PCR level was carried out using ABI PRISM 7500 detection system (American Applied Biosystems, Bedford, MA, USA). Primer-Premier software (Premier-Biosoft, San Francisco, CA, USA) was used to design specific primers and synthesized by Invitrogen (Shanghai, China). The PCR primers were designed with the 6.0 version of oligonucleotide primer analysis software and synthesized by Invitrogen (Shanghai, China). For each gene, the 2^−Δ*ΔCt*^ method was used to determine the relative expression level of steady-state mRNA in the sample, and the results were normalized to the mean of GAPDH and β-actin.

### Determination of Heart NO Content and NOS Activities

The chicken hearts were homogenized on ice in physiological saline, centrifuged at 750 × g for 15 min at 4°C, and then the supernatant was collected. NO and NOS levels are markers of oxidative damage. The NO and NOS activity determination kit (Nanjing Jiancheng Bioengineering Research Institute, Nanjing, China) was used to determine the NO content and NOS activity. Methanol was used in this study, and the ELX800 microplate reader (BioTek Instruments, Inc., Winooski, VT, USA) was used to detect OD at 550 and 530 nm.

The NOS Activity Detection kit was used to measure the activity of iNOS and total NOS. The constitutive NOS activity was calculated by subtracting iNOS from the total NOS. The PCR primers (Table 1) were designed using oligonucleotide primer analysis software (version 6.0) and synthesized by Invitrogen (Shanghai, China).

**Table 1:** Specific amplification primers of NOS 1–3 mRNA genes.

**Gene**	**Primer sequences (5^**′**^-3^**′**^)**
eNOS P3	5**′**-CAGCCCCCGCTATTACTCCGT-3**′**
	5**′**-AGCCCAAAGATGTCCTCGTG-3**′**
eNOS P4	5**′**-CTCCTTCCTGGACATCACG-3**′**
	5**′**-CTGCGCCTCCTCCATCT-3**′**
iNOS	5**′**-CCTGGAGGTCCTGGAAGAGT-3**′**
	5**′**-CCTGGGTTTCAGAAGTGGC-3**′**
NNOS	5**′**-GTGGCTCTTAACAGAACTTCC-3**′**
	5**′**-TCCCATAAAGCAGCGAGA-3**′**

### Determination of the Nrf2 Signal Pathway Related Factor Expression Level (Nrf2\HO-1\NQO1)

The PCR primers (Table 2) were designed using oligonucleotide primer analysis software (version 6.0) and synthesized by Invitrogen (Shanghai, China).

**Table 2:** Primer sequences of Nrf2, HO-1, and NQO1.

**Gene**	**Primer sequences (5^**′**^-3^**′**^)**
Nrf2	5**′**-CCTTGTCCTTTGATGACTGC-3**′**
	5**′**-TGGGTGGCTGAGTTTGATTA-3**′**
HO-1	5**′**-CCCTGGTCTGTCTAATGTC-3**′**
	5**′**-AATCCAGACTCCCACCTAA-3**′**
NQO1	5**′**-GTTCAATGCCGTGCTCTCAC-3**′**
	5**′**-TCCCATAAAGCAGCGAGA-3**′**

### Statistical Analysis

The results are expressed as the mean ± standard deviation and analyzed with GraphPad Prism 5.0 (GraphPad Software, Inc., La Jolla, CA, USA) and SPSS 19.0 software (SPSS Inc., Chicago, Illinois, USA). Statistical analyses were performed using Student's *t*-test (GraphPad Software, Inc., La Jolla, CA, USA) and one-way ANOVA test followed by Tukey's *post-hoc* pairwise comparison. In the same column of data in the table, if the letters are the same, the difference is not significant (*p* > 0.05); if the letters are completely different, the difference is significant; lowercase letters indicate a significant level (*p* < 0.05); uppercase letters indicate an extremely significant level (*p* < 0.01). All data passed the equal variance test. The difference between the averages is multiple comparisons afterward by Tukey's honest significance test.

## Results

### Effects of Transport Stress on the Cardiac Performance of Newborn Chicks

**Figure 1:**
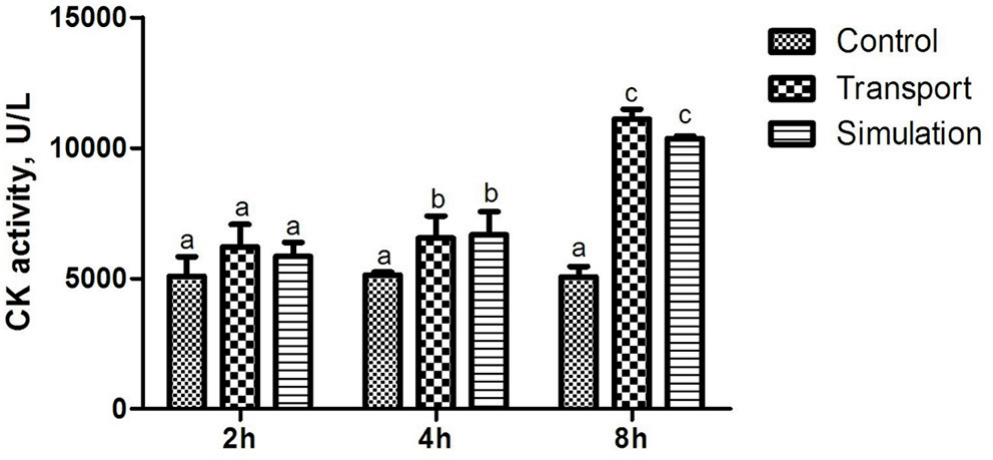
Effects of transport stress on the cardiac performance of newborn chicks. Row (Column) with different small letter superscripts indicates a significant difference (*p* < 0.05). Row (Column) with different small letter superscripts indicates no significant difference (*p* > 0.05). Control, the control groups; Transport, the transport groups; Simulation, the simulation transport groups.

The results of the determination of heart function (CK activity) of newborn chickens are shown in Figure 1. Compared with the control group, the CK activity in the transport group and the simulation group at the time of transportation was significantly changed, showing a significant difference (*p* < 0.05); There was no significant difference in CK activity among the three groups with a time of 2 h (*p* > 0.05), and the CK activity in the transport group and the simulation group with a transport time of 4 and 8 h demonstrated a significant difference compared to control group (*p* < 0.05).

### Cardiac Pathology Histological Observation

**Figure 2:**
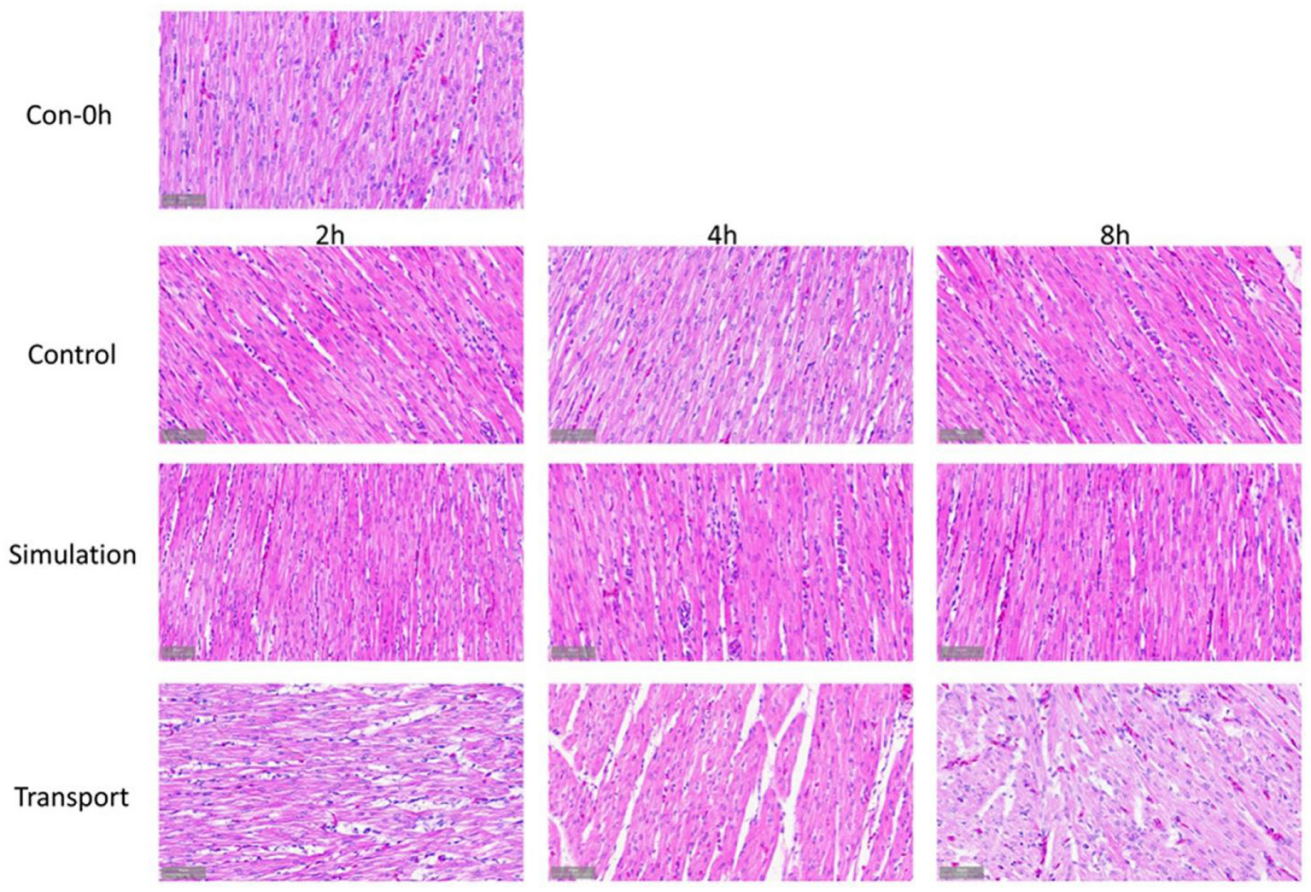
Comparison of macroscopic view from the chick heart in groups (400 × H&E staining).

Observing the histological changes of the heart through a microscope, it can be seen from the Figure 2 that at 0 h of the control group, the heart tissue structure is complete, the endocardium is intact and evenly distributed, the cardiomyocytes are arranged neatly, the cytoplasm and nucleus are stained normally, and there is no abnormal structure; At each time point, the staining of myocardial cells in the transport group and the simulated transport group became lighter. After 4 h of transportation, the cardiomyocytes began to be arranged disorderly, and a small amount of powder stained particles appeared in the cells; at 8 h of transportation, compared with the control group, part of the endocardium thickened, the diameter of the cardiomyocytes becomes larger, the staining is lighter, the cardiomyocytes edema is obvious, and a large number of powder-stained particles are seen in the cytoplasm. In the simulated transportation group, there was no significant change after simulated transportation for 2 h. The edema of myocardial cells was significantly reduced at 4 and 8 h. In the simulated transportation group at 4 h, powder stained particles were occasionally seen. At 8 h, a small amount of powder-stained particles could be seen in myocardial cells.

### Effect of Transport Stress on Antioxidant Function

#### Effect of Transport Stress on MDA Content

**Figure 3:**
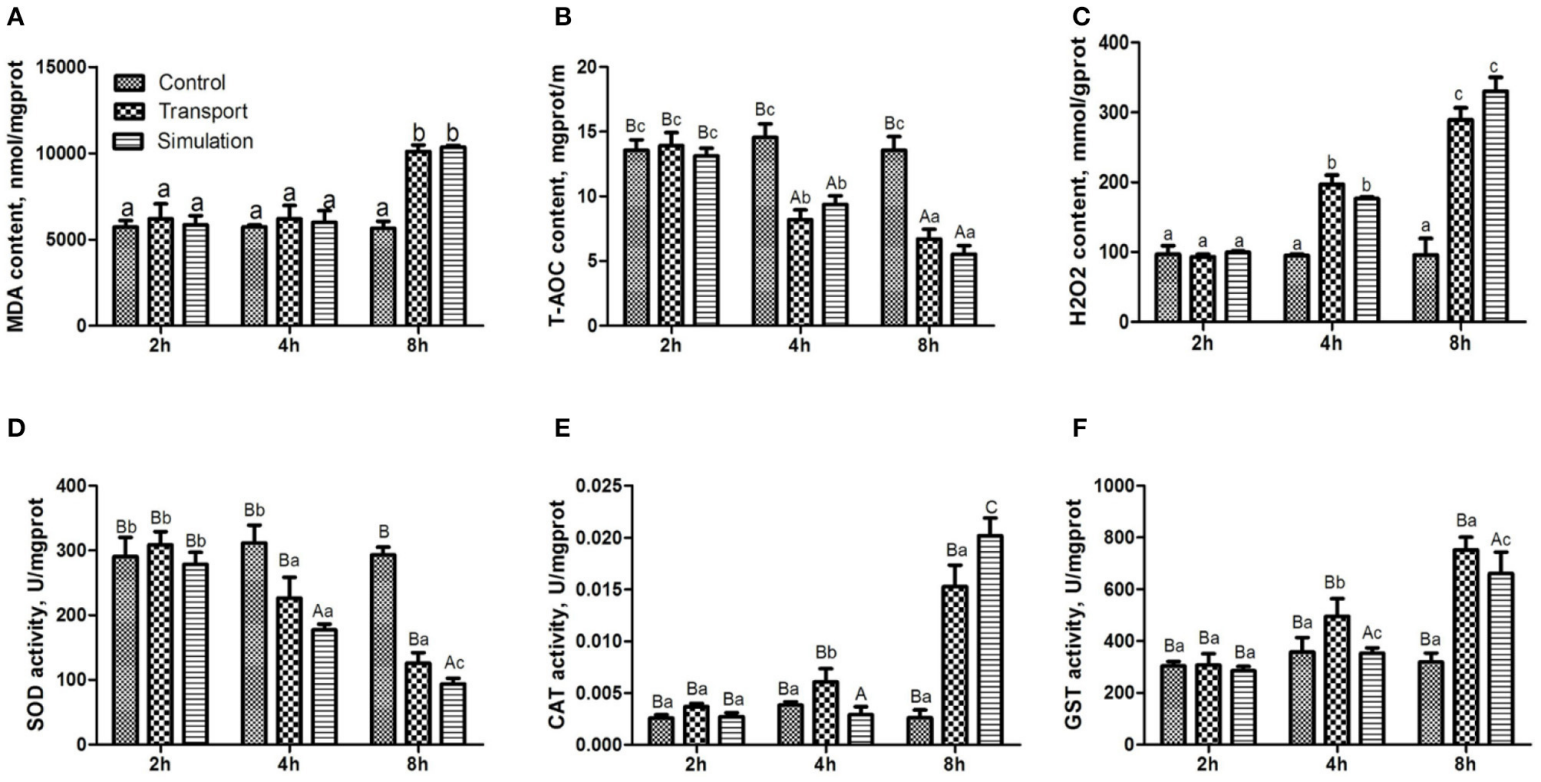
Effects of transport stress on antioxidant function and enzyme activity in the heart of newborn chicks. **(A)** Malondialdehyde (MDA) content, **(B)** total antioxidant capacity (T-AOC) content, **(C)** Hydrogen peroxide (H_2_O_2_) content, and **(D)** total superoxide dismutase (T-SOD) activity. **(E)** catalase (CAT) activity, **(F)** glutathione S-transferase (GST) activity. Data are presented as the mean ± SD. Compared with the control: Row (Column) with different capital letter superscripts indicates an extremely significant difference (*p* < 0.01); Row (Column) with different small letter superscripts indicates a significant difference (*p* < 0.05); Row (Column) with different small letter superscripts indicates no significant difference (*p* > 0.05). Control, the control groups; Transport, the transport groups; Simulation, the simulation transport groups; g prot, gram of protein; mg prot, milligram of protein.

The results of the determination of MDA in heart tissue of the control group, transport group and simulation group at various transportation times were illustrated in Figure 3A. Compared with the control group, there was no significant distinction of MDA content in the transport group and the simulation group at 2 and 4 h (*p* > 0.05). When the transportation time increased to 8 h, the MDA content in the transport group and simulation group increased significantly (*p* < 0.05); during the whole experiment, the control group showed no remarkable change in MDA content (*p* > 0.05).

#### Effect of Transport Stress on T-AOC Content

The results of the determination of T-AOC in the heart tissue of the different groups at various transportation time points were shown in Figure 3B. There was no conspicuous difference in the content of T-AOC among the three groups at 2 h (*p* > 0.05). With the prolongation of transport stress time, the T-AOC content in the transport group and simulation group showed a downward trend, compared with the control group at that moment (*p* < 0.01).

#### Effect of Transport Stress on H_2_O_2_ Content

As can be seen from Figure 3C, there was no difference in the content of H_2_O_2_ among the three groups at 2 h (*p* > 0.05). With the extension of the transportation time, the H_2_O_2_ content was significantly increased in the transport group and simulation group at 4 and 8 h (*p* < 0.01).

#### Effect of Transport Stress on Antioxidant Enzyme Activity

Figure 3D shows that the SOD enzyme activity of the transport group and simulation group is decreasing. Compared with the control group, the SOD levels in the transport group and simulation group after 4 h of stress are significantly decreased (*p* < 0.05), and the difference between the transport group and simulation group was extremely significant (*p* < 0.01) at 8 h. The CAT content and GST content in the transport group at 4 h were significantly increased (*p* < 0.01; Figures 3E,F). The CAT content and GST content in the transport group and simulation group at 8 h were obviously increased (*p* < 0.01; Figures 3E,F).

### Effect of Transport Stress on NO Metabolism (NO-NOS System)

#### Effects of Transport Stress on NO Content

**Figure 4:**
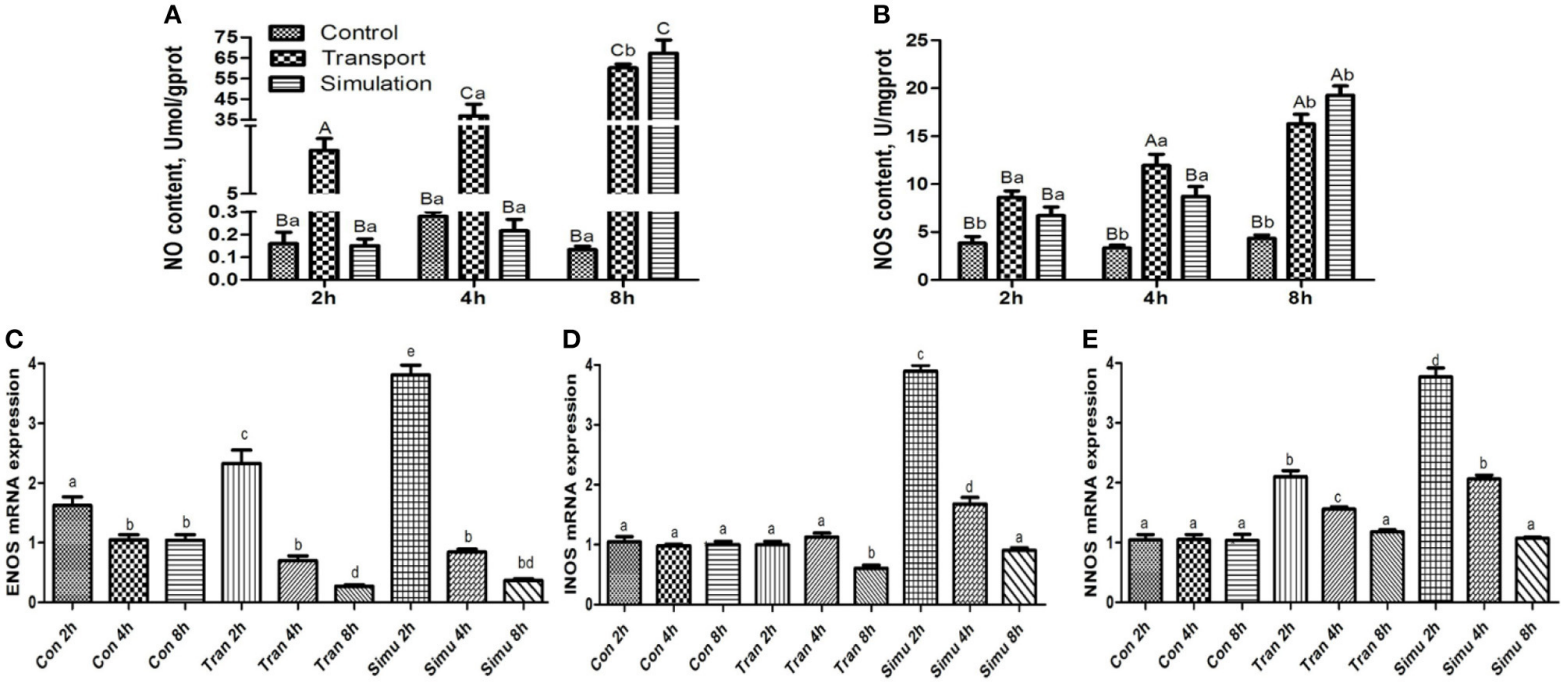
Effect of transport stress on NO metabolism (NO- NOS system) in the heart of newborn chicks. **(A)** NO content, **(B)** NOS activity, **(C)** the expression of ENOS mRNA at 2, 4, and 8 h, **(D)** the expression of INOS mRNA at 2, 4, and 8 h, and **(E)** the expression of NNOS mRNA at 2,4, and 8 h. Data are presented as the mean ± SD. Compared with the control: Row (Column) with different capital letter superscripts indicates an extremely significant difference (*p* < 0.01); Row (Column) with different small letter superscripts indicate significant difference (*p* < 0.05); Row (Column) with different small letter superscripts indicate no significant difference (*p* > 0.05). Con, the control groups; Tran, the transport groups; Simu, the simulation transport groups; mgprot, milligram of protein.

As can be seen in Figure 4A, the NO content in the transport group and the simulation group showed an upward trend. Compared with the control group in the same period, the NO content in the experimental group and the 8-h simulation group at each period increased significantly (*p* < 0.01). The difference between NO content in the experimental group and the simulation group at 2 and 4 h was very significant (*p* < 0.01).

#### Effect of Transport Stress on NOS Activity

As can be seen in Figure 4B illustrates the findings of the NOS content of the experimental group and the simulation group showed an upward trend with the increase of transport time. Compared with the control group at the same period, the NOS content of the experimental group and the simulation group increased significantly (*p* < 0.05), while the NOS content in the experimental group and the simulation group increased significantly at 4 and 8 h (*p* < 0.01).

#### Effect of Transport Stress on the mRNA Expression of NOS 1–3

Compared with the control group, the mRNA expression levels of the three genes in the transport group and the simulation group showed a downward trend as the stress time increased (Figures 4C–E).

### Effect of Transport Stress on Nrf2/HO-1/NQO1 Signaling Pathway

**Figure 5:**
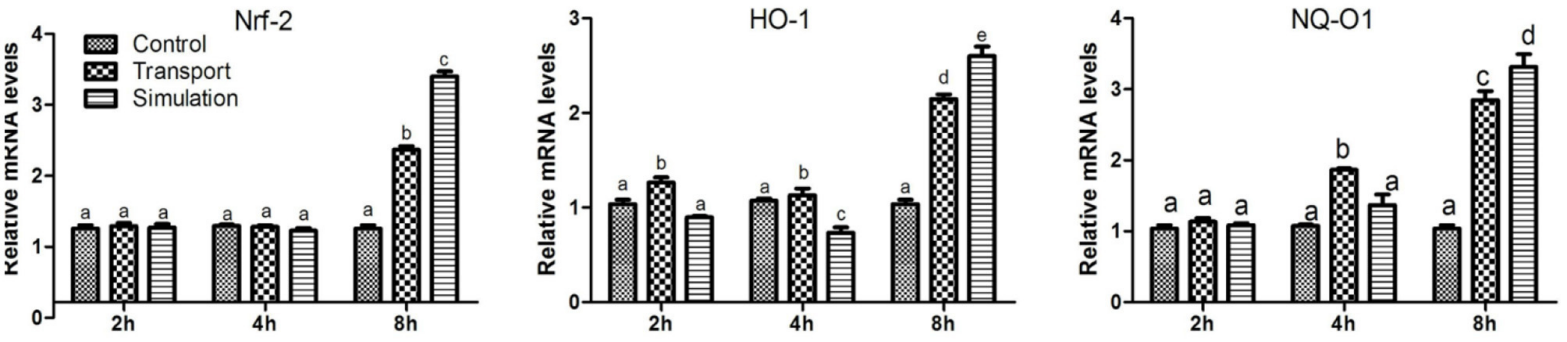
Effect of transport stress on Nrf2/HO-1/NQO1 signaling pathway in heart tissue of newborn chicks (the expression of Nrf2/HO-1/NQO1 mRNA). Data are presented as the mean ± SD. Compared with the control: Row (Column) with different small letter superscripts indicates a significant difference (*p* < 0.05); Row (Column) with different small letter superscripts indicates no significant difference (*p* > 0.05). Control, the control groups; Transport, the transport groups; Simulation, the simulation transport groups.

As shown in Figure 5, compared with the control group, the levels of Nrf2 mRNA in the transport group and the simulation group increased significantly at 8 h (*p* < 0.01), however, the mRNA expression of Nrf2 was no significant difference at 2 and 4 h (*p* > 0.05). Similarly, the results showed that compared with the control group, the mRNA levels of NQO1 and HO-1 increased in a dose-dependent manner. The difference was significant in the 2 h group (*p* < 0.05), and the difference was significant in the 4 and 8 h groups (*p* < 0.01).

## Discussion

For the poultry industry, transport stress is one of the unavoidable stresses. Transportation will cause stress to the chicks to varying degrees, such as mild discomfort, accelerated breathing, heartbeat, and body temperature rise (6, 17). The experimental animals themselves will suffer from the insufficient microcirculation blood supply, hypoxia, and even exhaustion and death. Transport stress also reduces the animal's live weight (18). The existing literature shows that the weight of all birds after transportation is significantly lower than before (19). Research by Gregory and Austin found that the mortality rate of broilers during transportation to the processing plant was 0.19% (17). A large number of statistics show that the average mortality rate of broilers during transportation is 0.46% (20).

In our current study, the weight loss of chicks with longer transport stress is greater, which is consistent with previous studies (6). However, the impact mechanism of transport stress on the weight loss and heart function damage of newborn chicks have not been fully elucidated. Transport stress exerts its regulatory and toxic effects on the animal body by regulating the antioxidant function of the heart (21), the metabolism of NO and the expression of NOS 1-3 mRNA (4), and induces oxidative stress to increase the level of Nrf2 *via* activating the Nrf2 signal pathway (5), and ultimately lead to chicks weight loss and heart damage.

CK kinase exists in the cytoplasm or mitochondria of the heart, and it is an important kinase that promotes ATP regeneration, muscle contraction, and energy transfer (22–24). The increase of CK activity in the blood is one of the important characteristics of animals under stress, reflecting the damage of related tissue cells and the degree of damage (24, 25). When chickens are under heat stress, their food intake is reduced, resulting in lower glycogen levels and an insufficient supply of muscle energy, eventually leading to muscle wasting illness, such as muscular dystrophy (26, 27). Therefore, CK in the muscles escapes and the blood concentration increases. Fasting stress for 4–8 h increases the severity of cardiac damage. It is speculated that the significant increase in the content of CK and other enzymes in the blood leads to alterations in the vivo environment. Previous studies have shown that transport stress may cause heart damage to pigs (28), and simulated transportation significantly damaged the heart of rats (29). Transport stress may trigger acute heart failure, which may be an explanation for the sudden death of pigs during transportation (30, 31). According to previous studies, our results showed that CK activity of chicks was significantly increased after transport, indicating significant damage to the heart of the chicks. In addition, in this study, the transport stress showed a clear time-dependent on the CK activity detected in chick serum.

In addition to changes in production performance after transportation of livestock and poultry, there will be further oxidative stress (32). Studies have shown that the MDA content in camel plasma was increased after transportation, and it increased significantly after 24 h (33). Likewise, the concentration of MDA in the bovine serum was increased significantly after transportation, but the T-AOC level was decreased significantly (34). This study also confirmed that transport stress could cause a significant increase in serum MDA content and a significant decrease in T-AOC content in newborn chicks, indicating that transportation induced oxidative stress in chicks. Cold stress can reduce the activity of SOD in the lungs of chicks, increase the content of MDA in the tissues, and cause oxidative damage to lung DNA (35). Heat stress can increase the content of lipid peroxidation in the liver (36), significantly increase the activity of SOD in the liver, but decrease the activity of SOD in the kidneys (37). In this study, under the conditions of transport stress, the content of SOD in chicks showed a downward trend, while the contents of CAT and GST showed an upward trend. This further indicated that transport stress can disrupt the balance of oxidative and antioxidant systems. The oxidative system, protein oxidation and lipid peroxidation cause oxidative damage to organs (38). It is worth noting that in this study, in addition to setting up the experimental group for natural transportation, we also conducted a simulated transportation stress test by means of a shaking table. It can be seen from the results that when the transportation time lasted 8 h, the content of CAT and SOD significantly altered between the transport group and simulation group. There is a certain difference in the content of antioxidant enzymes in chicks under simulated transportation and natural transportation conditions, which further shows that transportation stress is a stress response caused by a complex stressor. In the actual transportation process, the animal body is affected by other than bumps. The common influence of many factors causes changes in various physiological functions (39). We have found that exposure to transport stress can increase the levels of oxidation products, and inhibit the activity of antioxidant molecules/enzymes, causing oxidative stress, and activating other defense mechanisms to resist this stress.

NO has significant biological activity and participates in the physiological and pathological processes of many systems such as circulation, respiration, immunity, nerves, and digestion (40, 41). Stressors such as local heating and cold exposure may stimulate NO production. NO is a highly reactive and unstable free radical gas (42). It generates NOS by oxidizing L-arginine and nicotinamide adenine dinucleotide phosphate (NADPH) as electron donors for citrulline (16). NOS subtypes include nNOS, iNOS, and eNOS. nNOS and eNOS are constitutively expressed in the cardiovascular system (43). eNOS is more common in coronary blood vessels and endocardial endothelial cells, and has a protective effect on cardiomyocytes (44, 45). nNOS plays a pivotal part in the myocardial sarcoplasmic reticulum, Golgi apparatus, mitochondria, and sarcolemma (46, 47). Although iNOS may be induced under certain stress conditions, but it is usually not expressed in the myocardium, and it is considered harmful to the heart (48). Studies have shown that cold stress could significantly increase the expression of iNOS mRNA in the hypothalamus of rats (49), indicating that iNOS in the hypothalamus is involved in the damage induced by cold stress (50). There was no significant change in the expression level of NOS l in the hypothalamus, while the expression of NOS2 and NOS3 was significantly increased, and the expression of NOS1 and NOS3 in the adrenal glands was significantly increased, but there was no expression of NOS2, indicating that different types of NOS and NOS in different tissues were caused by the damage to the body. Under transportation pressure, the NO content and NOS activity in the heart of chicks was increased, while the expression level of NOS 1-3 mRNA was decreased, indicating that transportation pressure could induce alterations in NO metabolism and the expression of NOS 1-3 mRNA, which in turn affects chicks. These results indicated that transport stress increased NO production by enhancing the activity of NOS and the transcription of NOS isoforms.

Nrf2, a transcription factor, is considered to be oxidative stress and cellular defense effective chemical regulator (51, 52). The Nrf2 signaling pathway is an important endogenous anti-oxidative stress pathway that exists in cells (53). It mainly activates antioxidant genes and expresses antioxidant proteins, changing the redox state, and maintaining intracellular balance (54). Nrf2 acts as an antioxidant and antidote by regulating the synthesis of GSH to eliminate ROS (heterologous metabolites) (55). And target gene expression, such as NQO1, GST and HO-1 subunits (56). NQO1 is one of the main antioxidant enzymes (57). As a homodimer, NQO1 needs to catalyze the reduction of quinone and semiquinone to stabilize hydroquinone and requires nicotinamide adenine dinucleotide or NADPH (58). Jo et al. found that coptisine could reduce oxidative stress damage in Chinese hamster lung fibroblasts (59). With the increase of Nrf2 protein expression and nuclear translocation, the antioxidant protein expression of HO-1 regulated by Nrf2 was significantly increased (60). NQO1 can also detoxify the NAPQI produced by the CYP2E1 enzyme from acetaminophen (61). The enzyme is responsible for catalyzing synthesis in GCL cells (62). Another key antioxidant enzyme HO-1 is involved in the degradation of the antioxidant heme, thereby producing antioxidants (63). Studies have found that propargyl amphetamine induced Nrf2 through extracellular signal-regulated kinase and phosphatidylinositol-3-kinase/protein kinase B signaling pathway when l-methyl-4-phenylpyridinium ion caused oxidative stress (64). The expression level and nuclear translocation of Nrf2 increased the expression of NQO1 protein. After inhibiting Nrf2 by siRNA, there was no increase in NQO1 expression (65). The above results confirmed that Nrf2 up-regulated the expression of NQO1 and played a role in resisting oxidative stress damage. In this study, the levels of Nrf2, HO-1 and NQO1 mRNA in the heart of chicks were increased in a dose-dependent manner after transport stress treatment, confirming that transport stress could increase Nrf2 levels by inducing oxidative stress and activating the Nrf2 signaling pathway. In short, transport stress-induced NO-NOS system metabolic disorders and heart oxidative stress are mitigated by activating the Nrf2/HO-1/NQO1 antioxidant defense response in newborn chicks.

## Data Availability Statement

The raw data supporting the conclusions of this article will be made available by the authors, without undue reservation.

## Ethics Statement

The animal study was reviewed and approved by Institutional Animal Care and Use Committee of Northeast Agricultural University and the animal welfare regulations. Written informed consent was obtained from the owners for the participation of their animals in this study.

## Author Contributions

J-LL, H-LX, HL, and R-KB: funding acquisition and writing—original draft. H-LX, HL, R-KB, AE, and HG: visualization. H-LX, HL, and R-KB: analyzed the data. HL, R-KB, AE, and HG: methodology and writing—review and editing. All authors read and approved the final manuscript.

## Funding

This study has received assistance from Distinguished Professor of Longjiang Scholars Support Project (No. T201908), China Agriculture Research System of MOF and MARA (No. CARS-35), and Heilongjiang Touyan Innovation Team Program.

## Conflict of Interest

The authors declare that the research was conducted in the absence of any commercial or financial relationships that could be construed as a potential conflict of interest.

## Publisher's Note

All claims expressed in this article are solely those of the authors and do not necessarily represent those of their affiliated organizations, or those of the publisher, the editors and the reviewers. Any product that may be evaluated in this article, or claim that may be made by its manufacturer, is not guaranteed or endorsed by the publisher.

## References

[1] Miranda-de la Lama GC Rodriguez-Palomares M Cruz-Monterrosa RG Rayas-Amor AA Pinheiro RSB Galindo FM. Long-distance transport of hair lambs: effect of location in pot-belly trailers on thermo-physiology, welfare and meat quality. Trop Anim Health Prod. (2018) 50:327–36. 10.1007/s11250-017-1435-0 28963625

[2] Schnyder P Schonecker L Schupbach-Regula G Meylan M. Effects of management practices, animal transport and barn climate on animal health and antimicrobial use in swiss veal calf operations. Prev Vet Med. (2019) 167:146–57. 10.1016/j.prevetmed.2019.03.007 30948232

[3] Rigby CE Pettit JR Baker MF Bentley AH Salomons MO Lior H. Flock infection and transport as sources of salmonellae in broiler chickens and carcasses. Can J Comp Med. (1980) 44:328–37. 7000322 PMC1320082

[4] Sun F Zuo YZ Ge J Xia J Li XN Lin J. Transport stress induces heart damage in newly hatched chicks via blocking the cytoprotective heat shock response and augmenting nitric oxide production. Poult Sci. (2018) 97:2638–46. 10.3382/ps/pey146 29750253

[5] Ge J Li H Sun F Li XN Lin J Xia J. Transport stress-induced cerebrum oxidative stress is not mitigated by activating the Nrf2 antioxidant defense response in newly hatched chicks. J Anim Sci. (2017) 95:2871–8. 10.2527/jas2017.1559 28727098

[6] Li ZY Lin J Sun F Li H Xia J Li XN. Transport stress induces weight loss and heart injury in chicks: disruption of ionic homeostasis via modulating ion transporting atpases. Oncotarget. (2017) 8:24142–53. 10.18632/oncotarget.15903 28445983 PMC5421834

[7] Bao F Tao L Zhang H. Neuroprotective effect of natural alkaloid fangchinoline against oxidative glutamate toxicity: involvement of keap1-Nrf2 axis regulation. Cell Mol Neurobiol. (2019) 39:1177–86. 10.1007/s10571-019-00711-6 31270710 PMC11452225

[8] Su X Jiang X Meng L Dong X Shen Y Xin Y. Anticancer activity of sulforaphane: the epigenetic mechanisms and the Nrf2 signaling pathway. Oxid Med Cell Longev. (2018) 2018:5438179. 10.1155/2018/5438179 29977456 PMC6011061

[9] Lin X Bai D Wei Z Zhang Y Huang Y Deng H. Curcumin attenuates oxidative stress in raw264.7 cells by increasing the activity of antioxidant enzymes and activating the Nrf2-Keap1 pathway. PLoS ONE. (2019) 14:e0216711. 10.1371/journal.pone.0216711 31112588 PMC6528975

[10] Ruiz S Pergola PE Zager RA Vaziri ND. Targeting the transcription factor Nrf2 to ameliorate oxidative stress and inflammation in chronic kidney disease. Kidney Int. (2013) 83:1029–41. 10.1038/ki.2012.439 23325084 PMC3633725

[11] Zheng X Wang G Bin P Meng T Niu Y Yang M. Time-course effects of antioxidants and phase II enzymes on diesel exhaust particles-induced oxidative damage in the mouse lung. Toxicol Appl Pharmacol. (2019) 366:25–34. 10.1016/j.taap.2019.01.010 30641076

[12] Abdul-Ghani S Yanai J Abdul-Ghani R Pinkas A Abdeen Z. The teratogenicity and behavioral teratogenicity of Di(2-ethylhexyl) phthalate (Dehp) and Di-butyl phthalate (Dbp) in a Chick Model. Neurotoxicol Teratol. (2012) 34:56–62. 10.1016/j.ntt.2011.10.001 22019469

[13] Mann GE. Nrf2-mediated redox signalling in vascular health and disease. Free Radic Biol Med. (2014) 75 (Suppl. 1):S1. 10.1016/j.freeradbiomed.2014.10.595 26461277

[14] Fan W Liu Q Zhu X Wu Z Li D Huang F. Regulatory effects of anesthetics on nitric oxide. Life Sci. (2016) 151:76–85. 10.1016/j.lfs.2016.02.094 26946305

[15] Li XN Zuo YZ Qin L Liu W Li YH Li JL. Atrazine-xenobiotic nuclear receptor interactions induce cardiac inflammation and endoplasmic reticulum stress in quail (coturnix coturnix coturnix). Chemosphere. (2018) 206:549–59. 10.1016/j.chemosphere.2018.05.049 29778080

[16] Forstermann U Sessa WC. Nitric oxide synthases: regulation and function. Eur Heart J. (2012) 33:829–37:37a–d. 10.1093/eurheartj/ehr304 21890489 PMC3345541

[17] Vecerek V Grbalova S Voslarova E Janackova B Malena M. Effects of travel distance and the season of the year on death rates of broilers transported to poultry processing plants. Poult Sci. (2006) 85:1881–4. 10.1093/ps/85.11.1881 17032817

[18] Damtew A Erega Y Ebrahim H Tsegaye S Msigie DJ. The effect of long distance transportation stress on cattle: a review. Biomed J Sci Tech Res. (2018) 2:5. 10.26717/BJSTR.2018.03.000908

[19] Menon DG Bennett DC Schaefer AL Cheng KMJ. Transportation stress and the incidence of exertional rhabdomyolysis in emus (*Dromaius novaehollandiae*). Poultry Sci. (2014) 93:273–84. 10.3382/ps.2013-03260 24570448

[20] Grilli C Stocchi R Loschi AR Conti F Rea S. Survey on broiler pre-slaughter mortality in a commercial abattoir of central Italy. Ital J Food Saf. (2018) 7:5878. 10.4081/ijfs.2018.5878 30538957 PMC6240839

[21] Azad MB Chen Y Gibson SB. Regulation of autophagy by reactive oxygen species (Ros): implications for cancer progression and treatment. Antioxid Redox Signal. (2009) 11:777–90. 10.1089/ars.2008.2270 18828708

[22] Wallimann T Tokarska-Schlattner M Schlattner U. The creatine kinase system and pleiotropic effects of creatine. Amino Acids. (2011) 40:1271–96. 10.1007/s00726-011-0877-3 21448658 PMC3080659

[23] Guimaraes-Ferreira L. Role of the phosphocreatine system on energetic homeostasis in skeletal and cardiac muscles. Einstein. (2014) 12:126–31. 10.1590/S1679-45082014RB2741 24728259 PMC4898252

[24] Baird MF Graham SM Baker JS Bickerstaff GF. Creatine-kinase- and exercise-related muscle damage implications for muscle performance and recovery. J Nutr Metab. (2012) 2012:960363. 10.1155/2012/960363 22288008 PMC3263635

[25] Chulayo AY Muchenje V. The effects of pre-slaughter stress and season on the activity of plasma creatine kinase and mutton quality from different sheep breeds slaughtered at a smallholder abattoir. Asian Australas J Anim Sci. (2013) 26:1762–72. 10.5713/ajas.2013.13141 25049767 PMC4092882

[26] Wasti S Sah N Mishra B. Impact of heat stress on poultry health and performances, and potential mitigation strategies. Animals. (2020) 10:1266. 10.3390/ani10081266 32722335 PMC7460371

[27] Zaboli G Huang X Feng X Ahn DU. How can heat stress affect chicken meat quality?–a review. Poult Sci. (2019) 98:1551–6. 10.3382/ps/pey399 30169735

[28] Munzel T Sorensen M Schmidt F Schmidt E Steven S Kroller-Schon S. The adverse effects of environmental noise exposure on oxidative stress and cardiovascular risk. Antioxid Redox Signal. (2018) 28:873–908. 10.1089/ars.2017.7118 29350061 PMC5898791

[29] Arts JW Kramer K Arndt SS Ohl F. The impact of transportation on physiological and behavioral parameters in wistar rats: implications for acclimatization periods. ILAR J. (2012) 53:E82–98. 10.1093/ilar.53.1.82 23382273

[30] Rioja-Lang FC Brown JA Brockhoff EJ Faucitano L. A review of swine transportation research on priority welfare issues: a Canadian perspective. Front Vet Sci. (2019) 6:36 10.3389/fvets.2019.00036 30854374 PMC6395376

[31] Bao E Sultan KR Nowak B Hartung J. Expression and distribution of heat shock proteins in the heart of transported pigs. Cell Stress Chaperones. (2008) 13:459–66. 10.1007/s12192-008-0042-4 18465207 PMC2673930

[32] Deters E Hansen SL. Invited review: linking road transportation with oxidative stress in cattle and other species. Appl Anim Sci. (2020) 36:183–200. 10.15232/aas.2019-01956

[33] Nazifi S Saeb M Baghshani H Saeb S. Influence of road transportation during hot summer conditions on oxidative status biomarkers in Iranian dromedary camels (*Camelus dromedarius*). Afr J Biochem Res. (2009) 3:282–7. 10.5897/AJBR.9000143

[34] Chirase NK Greene LW Purdy CW Loan RW Auvermann BW Parker DB. Effect of transport stress on respiratory disease, serum antioxidant status, and serum concentrations of lipid peroxidation biomarkers in beef cattle. Am J Vet Res. (2004) 65:860–4. 10.2460/ajvr.2004.65.860 15198229

[35] Wei H Zhang R Su Y Bi Y Li X Zhang X. Effects of acute cold stress after long-term cold stimulation on antioxidant status, heat shock proteins, inflammation and immune cytokines in broiler heart. Front Physiol. (2018) 9:1589. 10.3389/fphys.2018.01589 30483152 PMC6243113

[36] Das A. Heat stress-induced hepatotoxicity and its prevention by resveratrol in rats. Toxicol Mech Methods. (2011) 21:393–9. 10.3109/15376516.2010.550016 21426263

[37] Akbarian A Michiels J Degroote J Majdeddin M Golian A De Smet S. Association between heat stress and oxidative stress in poultry; mitochondrial dysfunction and dietary interventions with phytochemicals. J Anim Sci Biotechnol. (2016) 7:37. 10.1186/s40104-016-0097-5 27354915 PMC4924307

[38] Lobo V Patil A Phatak A Chandra N. Free radicals, antioxidants and functional foods: impact on human health. Pharmacogn Rev. (2010) 4:118–26. 10.4103/0973-7847.70902 22228951 PMC3249911

[39] Yaribeygi H Panahi Y Sahraei H Johnston TP Sahebkar A. The impact of stress on body function: a review. Excli J. (2017) 16:1057–72. 10.17179/excli2017-480 28900385 PMC5579396

[40] Rosselli M Keller R Dubey RK. Role of nitric oxide in the biology, physiology and pathophysiology of reproduction. Hum Reprod Update. (1998) 4:3–24. 10.1093/humupd/4.1.3 9622410

[41] Kuo PC Schroeder RA. The emerging multifaceted roles of nitric oxide. Ann Surg. (1995) 221:220–35. 10.1097/00000658-199503000-00003 7717775 PMC1234563

[42] Phaniendra A Jestadi DB Periyasamy L. Free radicals: properties, sources, targets, and their implication in various diseases. Indian J Clin Biochem. (2015) 30:11–26. 10.1007/s12291-014-0446-0 25646037 PMC4310837

[43] Forstermann U Li H. Therapeutic effect of enhancing endothelial nitric oxide synthase (Enos) expression and preventing Enos uncoupling. Br J Pharmacol. (2011) 164:213–23. 10.1111/j.1476-5381.2010.01196.x 21198553 PMC3174401

[44] Seddon M Shah AM Casadei B. Cardiomyocytes as effectors of nitric oxide signalling. Cardiovasc Res. (2007) 75:315–26. 10.1016/j.cardiores.2007.04.031 17568574

[45] Hsieh PC Davis ME Lisowski LK Lee RT. Endothelial-cardiomyocyte interactions in cardiac development and repair. Annu Rev Physiol. (2006) 68:51–66. 10.1146/annurev.physiol.68.040104.124629 16460266 PMC2754585

[46] Tengan CH Rodrigues GS Godinho RO. Nitric oxide in skeletal muscle: role on mitochondrial biogenesis and function. Int J Mol Sci. (2012) 13:17160–84. 10.3390/ijms131217160 23242154 PMC3546744

[47] Zhang YH. Nitric oxide signalling and neuronal nitric oxide synthase in the heart under stress. F1000Res. (2017) 6:742. 10.12688/f1000research.10128.1 28649367 PMC5464233

[48] Wilmes V Lux C Niess C Gradhand E Verhoff MA Kauferstein S. Changes in gene expression patterns in postmortem human myocardial infarction. Int J Legal Med. (2020) 134:1753–63. 10.1007/s00414-020-02311-2 32399898 PMC7417407

[49] Su Y Li S Xin H Li J Li X Zhang R. Proper cold stimulation starting at an earlier age can enhance immunity and improve adaptability to cold stress in broilers. Poult Sci. (2020) 99:129–41. 10.3382/ps/pez570 32416794 PMC7587771

[50] Strijdom H Chamane N Lochner A. Nitric oxide in the cardiovascular system: a simple molecule with complex actions. Cardiovasc J Africa. (2009) 20:303. 10.10520/EJC23284 19907806 PMC3721819

[51] Ma Q. Role of Nrf2 in oxidative stress and toxicity. Annu Rev Pharmacol Toxicol. (2013) 53:401–26. 10.1146/annurev-pharmtox-011112-140320 23294312 PMC4680839

[52] Ishii T Itoh K Takahashi S Sato H Yanagawa T Katoh Y. Transcription factor Nrf2 coordinately regulates a group of oxidative stress-inducible genes in macrophages. J Biol Chem. (2000) 275:16023–9. 10.1074/jbc.275.21.16023 10821856

[53] Petri S Körner S Kiaei M. Nrf2/ARE signaling pathway: key mediator in oxidative stress and potential therapeutic target in ALS. Neurol Res Int. (2012) 2012:878030. 10.1155/2012/878030 23050144 PMC3461296

[54] Winyard PG Moody CJ Jacob C. Oxidative activation of antioxidant defence. Trends Biochem Sci. (2005) 30:453–61. 10.1016/j.tibs.2005.06.001 15996871

[55] Vomund S Schäfer A Parnham MJ Brüne B Von Knethen A. Nrf2, the master regulator of anti-oxidative responses. Int J Mol Sci. (2017) 18:2772. 10.3390/ijms18122772 29261130 PMC5751370

[56] Lian F Wang XD. Enzymatic metabolites of lycopene induce Nrf2-mediated expression of phase ii detoxifying/antioxidant enzymes in human bronchial epithelial cells. Int J Cancer. (2008) 123:1262–8. 10.1002/ijc.23696 18566994 PMC3057886

[57] Ross D Siegel D. Functions of Nqo1 in cellular protection and Coq10 metabolism and its potential role as a redox sensitive molecular switch. Front Physiol. (2017) 8:595. 10.3389/fphys.2017.00595 28883796 PMC5573868

[58] Chen S Wu K Knox R. Structure-function studies of Dt-diaphorase (Nqo1) and Nrh: quinone oxidoreductase (Nqo2). Free Rad Biol Med. (2000) 29:276–84. 10.1016/S0891-5849(00)00308-7 11035256

[59] Jo H-G Park C Lee H Kim G-Y Keum Y-S Hyun JW. Inhibition of oxidative stress induced-cytotoxicity by coptisine in V79-4 chinese hamster lung fibroblasts through the induction of Nrf-2 mediated Ho-1 expression. Genes Genomics. (2020) 43:17–31. 10.1007/s13258-020-01018-3 33237503

[60] Loboda A Damulewicz M Pyza E Jozkowicz A Dulak J. Role of Nrf2/Ho-1 system in development, oxidative stress response and diseases: an evolutionarily conserved mechanism. Cell Mol Life Sci. (2016) 73:3221–47. 10.1007/s00018-016-2223-0 27100828 PMC4967105

[61] Moffit JS Aleksunes LM Kardas MJ Slitt AL Klaassen CD Manautou JE. Role of Nad (P) H: quinone oxidoreductase 1 in clofibrate-mediated hepatoprotection from acetaminophen. Toxicology. (2007) 230:197–206. 10.1016/j.tox.2006.11.052 17188792 PMC1885461

[62] Lu SC. Glutathione synthesis. Biochimica et Biophysica Acta General Subjects. (2013) 1830:3143–53. 10.1016/j.bbagen.2012.09.008 22995213 PMC3549305

[63] Gonzales S Pérez MJ Perazzo JC Tomaro ML. Antioxidant role of heme oxygenase-1 in prehepatic portal hypertensive rats. World J Gastroenterol. (2006) 12:4149. 10.3748/wjg.v12.i26.4149 16830363 PMC4087362

[64] Yang C Zhao J Cheng Y Le XC Rong J. N-propargyl caffeate amide (PACA) potentiates nerve growth factor (NGF)-induced neurite outgrowth and attenuates 6-hydroxydopamine (6-OHDA)-induced toxicity by activating the Nrf2/HO-1 pathway. ACS Chem Neurosci. (2015) 6:1560–9. 10.1021/acschemneuro.5b00115 26147318

[65] Rushworth SA MacEwan DJ O'Connell MAJTJoI. Lipopolysaccharide-induced expression of NAD (P) H: quinone oxidoreductase 1 and heme oxygenase-1 protects against excessive inflammatory responses in human monocytes. J Immunol. (2008) 181:6730–7. 10.4049/jimmunol.181.10.6730 18981090 PMC2923058

